# Inhalational versus intravenous maintenance of anesthesia for quality of recovery in patients undergoing corrective lower limb osteotomy: A randomized controlled trial

**DOI:** 10.1371/journal.pone.0247089

**Published:** 2021-02-19

**Authors:** Seung Hyun Kim, Hyang Mi Ju, Chong-Hyuck Choi, Hae Ri Park, Seokyung Shin

**Affiliations:** 1 Department of Anesthesiology and Pain Medicine, Anesthesia and Pain Research Institute, Severance Hospital, Yonsei University College of Medicine, Seoul, Korea; 2 Department of Anesthesiology and Pain Medicine, Anesthesia and Pain Research Institute, Yongin Severance Hospital, Yonsei University College of Medicine, Seoul, Korea; 3 Department of Orthopedic Surgery, Yonsei University College of Medicine, Seoul, Korea; 4 Department of Anesthesiology and Pain Medicine, Severance Hospital, Yonsei University College of Medicine, Seoul, Korea; Cleveland Clinic, UNITED STATES

## Abstract

**Background:**

Inhalational anesthesia and propofol-based total intravenous anesthesia (TIVA) are the two most popular methods of general anesthesia with distinct characteristics that may affect quality of recovery (QOR) differently. This study compared QOR after corrective lower limb osteotomy between desflurane-based inhalational anesthesia and propofol-based TIVA.

**Methods:**

Sixty-eight patients, ASA class I or II who underwent corrective lower limb osteotomy were randomized to receive either desflurane anesthesia or propofol TIVA. The primary outcome was quality of recovery 40 (QoR-40) questionnaire scores on postoperative day (POD) 1 and 2. Postoperative nausea scores, antiemetic requirements, and amount of opioid consumption via intravenous patient-controlled analgesia (IV PCA) were assessed as secondary outcomes.

**Results:**

Global QoR-40 scores on POD 1 (153.5 (140.3, 171.3) vs. 140.0 (120.0, 173.0), *P* = 0.056, 95% CI; -22.5, 0.2) and POD 2 (155.5 (146.8, 175.5) vs. 152.0 (134.0, 179.0), P = 0.209, 95% CI; -17.5, 3.9) were comparable between the two groups. Among the five dimensions of QoR-40, physical independence scores were significantly higher in the TIVA group compared to the Desflurane group on POD both 1 and 2. Nausea scores (0.0 (0.0, 0.0) vs. 1.0 (0.0, 3.5), *P* < 0.001) and number of patients requiring rescue antiemetics (0% vs. 15.2%, *P* = 0.017) were significantly lower in the TIVA group at the post anesthesia care unit (PACU). Although the number of bolus attempts between 0–24 h and the morphine equivalent dose of analgesics administered via IV PCA between 12–24 h were significantly less in the TIVA group compared to the Desflurane group, there was no significant difference between groups for the overall 48 h postoperative period.

**Conclusions:**

Propofol-based TIVA did not improve global QoR-40 scores compared with desflurane-based inhalational anesthesia. However, considering the better QoR-40 scores in the domain of physical independence and less nausea in the early postoperative period, propofol TIVA should be considered as a useful option in patients undergoing corrective lower limb osteotomy.

## Introduction

General anesthesia and surgery often cause discomfort in various aspects of the patient’s life. Although usually not life-threatening, delayed recovery after surgery not only reduces patient satisfaction, but is also undesirable in terms of efficient medical resource allocation and cost-effectiveness [1]. As an essential part of the surgical procedure, method of anesthesia should also be chosen and carefully planned to provide high-quality recovery and help patients return to normal daily activities faster.

Inhalational anesthesia and propofol-based total intravenous anesthesia (TIVA) are the two most used general anesthesia techniques. Previous studies that compared quality of recovery (QOR) after surgery under general anesthesia with these two methods report inconsistent results [2–8]. This may be due to the anesthetic agents having a varied degree of effects depending on type of surgery and patient population, and amount of surgical stress and inflammation [9–11].

Conventional methods used to assess postoperative recovery such as pain, vital signs, duration of hospital stay, morbidity and mortality are fragmentary, and are often not sufficient to evaluate QOR in patients without significant pre-existing comorbidities [12]. On the other hand, the quality of recovery 40 questionnaire (QoR-40) which was developed in 2000 is widely used and has been validated as a suitable method of assessing postoperative QOR in various types of surgeries and anesthesia techniques [13]. Such assessment tools can be especially useful in younger patient populations undergoing lower limb surgery where functional recovery is of much importance. There is a growing demand of corrective lower limb surgical procedures in patients that are young and physically active due to various reasons including early stage osteoarthritis causing mild deformities and pain, and limb lengthening for leg length discrepancy or idiopathic short stature [14,15]. These patients have a wide range of expectations encompassing pain relief, physical ability, and psychological well-being [16]. This study aimed to evaluate QOR between inhalational anesthesia with desflurane and TIVA with propofol in patients undergoing corrective lower limb osteotomies. As secondary outcomes, we also assessed postoperative analgesic requirements, nausea scores and antiemetic use after surgery.

## Materials and methods

### Ethics approval and patient selection

The protocol of the present study was approved by the Institutional Review Board and Hospital Research Ethics Committee of Yonsei University Health System, Seoul, South Korea (#4-2016-0164) on 15 April 2016, and registered at http://clinicaltrials.gov (NCT02826902). Written informed consent was obtained from all patients enrolled in this study, which was conducted from September 2016 to November 2019. The study population consisted of adult patients 19 years and older that underwent corrective lower limb osteotomy for either short stature, leg length discrepancy, or osteoarthritis. Patients with known allergies to propofol, heart disease (heart failure, myocardial infarction), previous major cardiovascular surgery, decreased renal function, recent stroke, or cognitive disorders were excluded.

### Interventions

Patients were randomly allocated to receive either desflurane anesthesia (Desflurane group) or propofol-based TIVA (TIVA group) on the day before surgery in a 1:1 ratio using a computer-generated random table generator by S. Shin. Due to significant differences in anesthetic technique, attending anesthesiologists were not able to be blinded to randomization. Patients and investigators in charge of administrating QoR-40 questionnaires remained blinded to group allocation. The investigator in charge of data analysis (S.H. Kim) remained blinded to group allocation until the entire analysis was completed. All surgical procedures were performed by one of two orthopaedic surgeons.

### Conduct of the study

Upon arrival at the operating room, standard monitoring including pulse oximetry, non-invasive blood pressure monitoring, electrocardiography, and bispectral index (BIS, VISTA Monitoring System, Aspect Medical Systems Inc., Norwood, MA, USA) monitoring were applied in all patients. In the Desflurane group, general anesthesia was induced with 5 mg/kg thiopental sodium and maintained with 4–7% desflurane and remifentanil infusion according to the Minto model [17]. In the TIVA group, anesthesia was induced and maintained with propofol and remifentanil using an effect-site target-controlled infusion pump (Orchestra Base Primea: Fresenius Vial, Brezins, France) according to the Marsh model [18] (propofol) and Minto [17] model (remifentanil). In all patients, the depth of general anesthesia was maintained at target BIS values between 40 and 60. Rocuronium (0.6 mg/kg) was used before intubation in all patients. Mechanical ventilation was performed with 8 ml/kg of tidal volume and respiratory rates were adjusted to maintain the end tidal CO_2_ levels between 35 to 45 mmHg with 50% oxygen/air mixture.

All patients received ramosetron 0.3 mg and fentanyl 1μg/kg at end of surgery. Concurrently, intravenous patient-controlled analgesia (IV PCA) consisting of fentanyl 0.2 μg/kg/ml and ramosetron 0.3 mg (total volume including normal saline, 150 ml) was initiated at a 2 ml/h (0.4 μg/kg/h) background infusion rate and 0.5 ml (0.1 μg/kg) on-demand bolus dose with a 15 min lockout time.

In addition to analgesics administrated via IV PCA, all patients were given 37.5 mg tramadol and 325 mg acetaminophen per oral twice daily as routine analgesic medications. Rescue analgesics consisted of either IV tramadol, acetaminophen, or meperidine were administered as needed.

### Data collection

QOR was assessed by using the QoR-40 questionnaire [19] at three time-points: the day before surgery, POD 1 and POD 2 in the evening on 7 pm. The QoR-40 is a 40-item questionnaire that has been validated in a diverse group of patients [13,19] and is comprised of five dimensions; physical comfort (12 items), emotional state (9 items), physical independence (5 items), psychological support (7 items), and pain (7 items). Each item is graded on a five-point Likert scale, and global QoR-40 scores range from 40 (extremely poor QOR) to 200 (excellent QOR). Nausea scores was evaluated at the PACU (highest score during the stay), POD 1 and POD 2 (same time point of QoR-40 assessment) using an 11-point verbal numerical rating scale (VNRS) ranging from 0 = no nausea to 10 = worst imaginable nausea. The incidence of vomiting and the amount of rescue antiemetics (metocloprimide 10mg or ramosetron 0.3mg) that were administered upon patient request were also recorded. The cumulative amount of analgesics administered, and number of bolus attempts via IV PCA up to 48 h after surgery were recorded and analyzed at 12h intervals. The number of patients requiring rescue analgesics up to postoperative 48h were assessed and compared between groups. Intraoperative heart rate, mean blood pressure, and BIS were recorded at baseline, 10 min after induction, cessation of anesthetics, and at tracheal extubation. Response time was defined as time taken from cessation of anesthetics to clear verbal response from the patient. Total intraoperative remifentanil dose was also recorded and compared between groups. Vital signs at the PACU were collected at admission and discharge.

### Statistical analysis

Sample size calculation was done under the hypothesis that a difference in global QoR-40 score of 10 or more between groups would be clinically significant. To obtain 90% power with a significance level of 5% by independent t-test and allowing for a dropout rate of 10%, 38 patients per group were needed.

A statistical analysis plan was drafted and outlined in our study protocol and was signed off before data analysis. Descriptive data are presented as median (interquartile range, IQR) for continuous variables, and number (proportion) for categorical variables. Continuous variables (e.g., QoR-40 questionnaire score, vital signs, time from cessation of anesthetics to verbal response, total remifentanil dose, VNRS for nausea, analgesics consumption via IV PCA) were checked for normality by using the Kolmogorov-Smirnov test, and analyzed by either the independent t-test or Mann-Whitney U test as indicated. Fisher’s exact test was used for the analysis of categorical variables such as the incidence of vomiting or use of antiemetics. Database lock was done by the primary investigator prior to final analysis. All statistical analyses were performed using SPSS Statistics version 25 (IBM Corp., Armonk, NY, USA).

## Results

### Patients and perioperative characteristics

**Fig 1:**
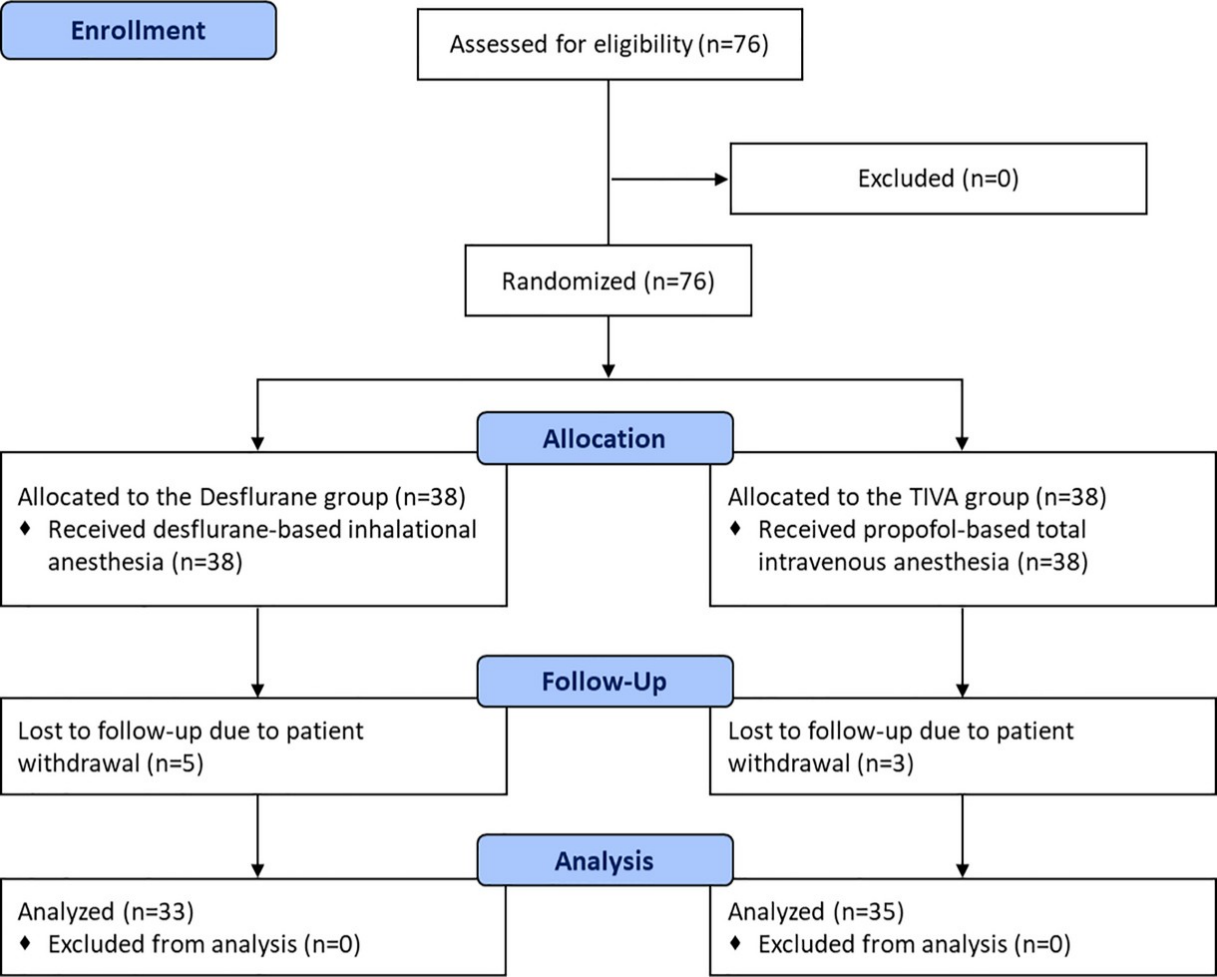
CONSORT flowchart of patient sample selection.

The CONSORT (Consolidated Standards of Reporting Trials) flow diagram is shown in Fig 1. Seventy-six patients were screened for eligibility, enrolled, and randomized to either the Desflurane group or the TIVA group. All randomized patients received surgery under allocated method of anesthesia. During postoperative follow-up, 5 and 3 patients in the Desflurane group and TIVA group, respectively, refused to further participate in completing the QoR-40 questionnaires and were dropped from the study. The remaining 68 patients (33 patients in the Desflurane group and 35 in the TIVA group) that received anesthesia as allocated and completed the QoR-40 questionnaires were included in the final analysis.

**Fig 2:**
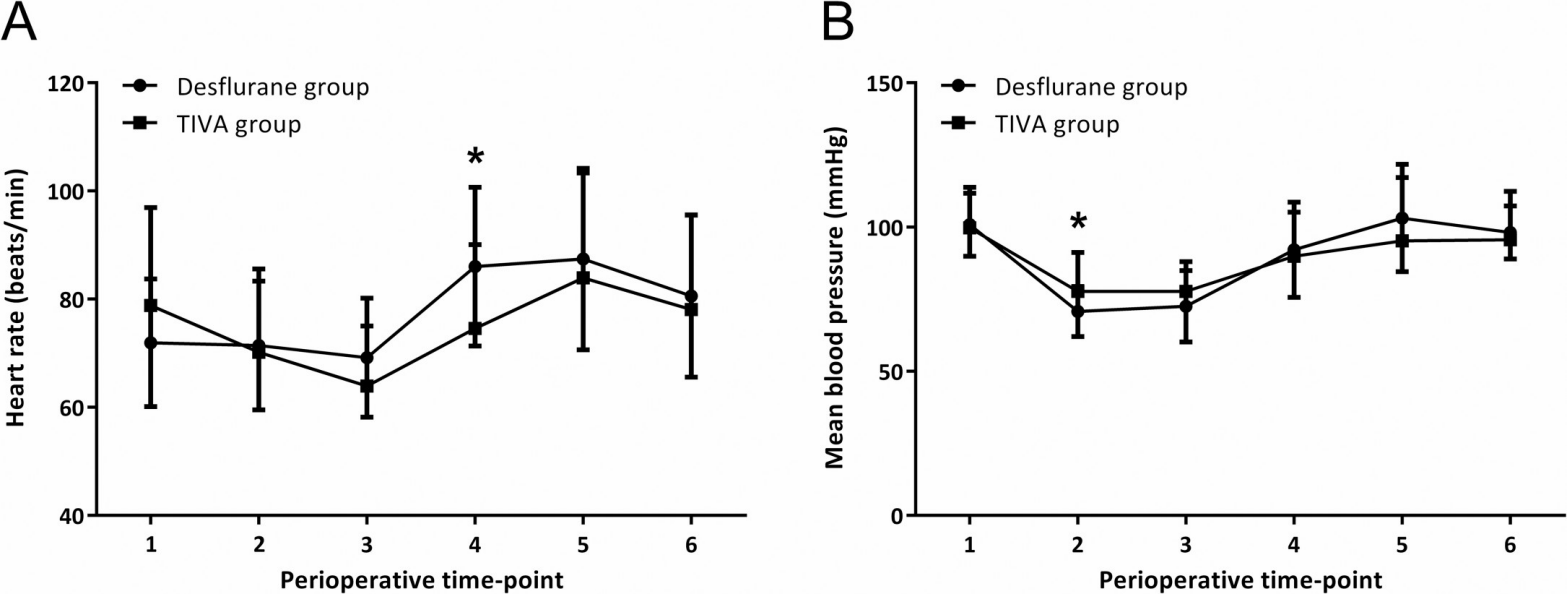
Perioperative (A) heart rate (beates/min) and (B) mean blood pressure (mmHg) of the Desflurane and TIVA group at six perioperative time-points; 1: at baseline before induction, 2: 10 min after anesthesia induction, 3: at cessation of anesthetics, 4: at tracheal extubation, 5: admission to post anesthesia care unit (PACU), 6: discharge from PACU. *P < 0.05 between two groups.

Patient characteristics are shown in Table 1. All demographic characteristics were similar between the two groups. Patients underwent lower limb lengthening or high tibial osteotomy due to either short stature, leg length discrepancy or osteoarthritis. The number of patients that presented with any pain before surgery was comparable between the two groups, and the preoperative VNRS pain scores in these patients ranged from 1 to 3. Intraoperative remifentanil usage was significantly greater in the TIVA group compared to the Desflurane group (0.12 (0.10, 0.15) μg/kg/min vs. 0.05 (0.04, 0.07) μg/kg/min, *P* < 0.001). Response time after cessation of anesthetics was significantly prolonged in the TIVA group compared to the Desflurane group (13.0 (7.9, 17.5) min vs. 8.3 (7.0, 10.0) min, *P* = 0.028). Perioperative vital signs are shown in Fig 2. Heart rate was significantly higher in the Desflurane group at tracheal extubation (73.0 (63.0, 82.0) bpm vs. 85.0 (75.0, 92.5) bpm, *P* = 0.001), and mean blood pressure was significantly lower in the Desflurane group at 10 min after induction (73.7 (69.3, 81.0) mmHg vs. 70.0 (66.2, 75.7) mmHg, *P* = 0.043).

**Table 1:** Patient characteristics.

	Desflurane group (n = 33)	TIVA group (n = 35)
Age (years)	40.0 (22.0, 53.5)	27.0 (22.0, 49.0)
Sex (male/female)	22/11	21/14
Height (cm)	163.6 (157.3, 173.5)	160.2 (156.8, 167.5)
Weight (kg)	65.0 (55.9, 73.0)	64.1 (55.0, 67.1)
BMI (kg/m^2^)	23.9 (21.8, 27.7)	23.8 (21.9, 27.1)
ASA physical status (I/II)	24/9	27/8
Etiology		
Short stature	18 (54.5)	23 (65.7)
Leg length discrepancy	0 (0.0)	2 (5.7)
Osteoarthritis	15 (45.5)	10 (28.6)
Patients with preoperative pain	14 (42.4)	11 (31.4)
Type of operation		
Lower limb lengthening	18 (54.5)	25 (71.4)
High tibial osteotomy	15 (45.5)	10 (28.6)
Surgeon who performed the operation (A/B)	18 (54.5) / 15 (45.5)	25 (71.4) / 10 (28.6)
Anesthesia duration (min)	230.0 (135.0, 277.5)	252.5 (141.0, 265.0)
Intraoperative fluid (mL)	750 (550, 990)	900 (650, 1150)
Intraoperative remifentanil dose (μg/kg/min)	0.05 (0.04, 0.07)	0.12 (0.10, 0.15)
Intraoperative propofol dose (mg)		1500.5 (1304.5, 2051.8)
Response time (min)	8.3 (7.0, 10.0)	13.0 (7.9, 17.5)

Data are presented as median (interquartile range) or number (proportion). TIVA, total intravenous anesthesia; BMI, body mass index; ASA, American Society of Anesthesiologists; Response time, time from cessation of anesthetics to verbal response from patient.

None of the patients experienced complications related to anesthesia. One patient in each group received correction surgery due to pin breakage and 3 patients in each group reported hypoesthesia in toes, feet, or heel at 2 months after surgery. Three and two patients experienced operative site infection and were treated with antibiotics in the Desflurane and TIVA group, respectively (*P* = 0.472).

### Quality of recovery and postoperative nausea and vomiting

Global scores and scores across the five different dimensions of the QoR-40 questionnaire are shown in Table 2. Although not statistically significant, an 11.1-point difference in mean global QoR-40 scores between groups was observed on POD 1 (153.5 (140.3, 171.3) vs. 140.0 (120.0, 173.0), *P* = 0.056, 95% CI; -22.5, 0.2). Global QoR-40 scores on POD 2 were comparable between the two groups (155.5 (146.8, 175.5) vs. 152.0 (134.0, 179.0), P = 0.209, 95% CI; -17.5, 3.9). Among the individual domains, the score for physical independence was significantly higher in the TIVA group compared to the Desflurane group both on POD 1 and 2. Other QoR-40 scores for individual domains were comparable between the two groups at all three time-points. In a single question assessing the presence of severe pain (Have you had severe pain in the last 24 hours?), the score was higher in the TIVA group compared to the Desflurane group on both POD 1 (3.0 (3.0, 4.0) vs. 3.0 (2.0, 3.0), P = 0.004) and 2 (4.0 (3.0, 4.0) vs. 3.0 (2.0, 3.5), P = 0.017).

**Table 2:** QoR-40 scores preoperatively and on postoperative days 1 and 2.

	Desflurane group (n = 33)	TIVA group (n = 35)	*P* value
Preoperative			
Emotional status	36.0 (29.0, 38.0)	34.0 (31.8, 39.3)	0.904
Physical comfort	50.0 (46.0, 57.0)	51.5 (44.5, 55.3)	0.694
Psychological support	30.0 (24.0, 32.0)	26.5 (24.8, 29.8)	0.649
Physical independence	24.0 (22.0, 25.0)	24.0 (21.8, 25.0)	0.653
Pain	26.0 (23.0, 33.0)	30.0 (22.8, 34.3)	0.113
Global QoR-40	167.0 (147.0, 178.0)	164.5 (150.5, 174.0)	0.578
POD 1			
Emotional status	33.0 (27.0, 42.0)	36.0 (31.0, 40.0)	0.100
Physical comfort	44.0 (36.0, 53.0)	44.5 (37.0, 52.0)	0.102
Psychological support	28.0 (25.0, 32.0)	27.0 (26.0, 29.0)	0.203
Physical independence	19.0 (14.0, 23.0)	20.5 (19.8, 24.0)	0.045
Pain	23.0 (20.0, 28.0)	26.0 (21.8, 28.3)	0.075
Global QoR-40	140.0 (120.0, 173.0)	153.5 (140.3, 171.3)	0.056
POD 2			
Emotional status	37.0 (30.0, 41.0)	36.5 (32.0, 41.3)	0.524
Physical comfort	48.0 (40.0, 56.0)	48.0 (43.3, 52.5)	0.551
Psychological support	28.0 (26.0, 33.0)	27.5 (25.8, 29.0)	0.367
Physical independence	19.0 (14.0, 21.0)	21.0 (18.8, 25.0)	0.033
Pain	25.0 (20.0, 26.0)	25.0 (21.8, 29.0)	0.085
Global QoR-40	152.0 (134.0, 179.0)	155.5 (146.8, 175.5)	0.209

Data are presented as median (interquartile range). TIVA, total intravenous anesthesia; QoR-40, quality of recovery-40; POD, postoperative day.

Nausea scores were significantly lower in the TIVA group compared to the Desflurane group at the PACU. (0.0 (0.0, 0.0) vs. 1.0 (0.0, 3.5), *P* < 0.001). None of the patients of the TIVA group required antiemetics at the PACU while 5 (15.2%) patients received rescue antiemetics in the Desflurane group (*P* = 0.017). There was no difference in PONV or the use of rescue antiemetics between groups during POD 1 and POD 2 (Table 3).

**Table 3:** Postoperative nausea and vomiting.

	Desflurane group (n = 33)	TIVA group (n = 35)	*P* value
PACU			
Nausea (VNRS)	1.0 (0.0, 3.5)	0.0 (0.0, 0.0)	< 0.001
Vomiting/retching	2 (6.1%)	0 (0.0%)	0.232
Use of rescue antiemetics	5 (15.2%)	0 (0.0%)	0.017
Postoperative period			
Nausea (VNRS)			
POD 1	2.0 (0.0, 4.0)	1.0 (0.0, 2.0)	0.094
POD 2	1.0 (0.0, 2.5)	0.0 (0.0, 2.0)	0.116
Vomiting/retching			
POD 1	4 (12.1%)	4 (11.4%)	> 0.999
POD 2	0 (0%)	0 (0%)	> 0.999
Use of rescue antiemetics			
POD 1	2 (6.1%)	4 (11.4%)	0.674
POD 2	0 (0%)	1 (2.9%)	> 0.999

Data are presented as median (interquartile range) or number (proportion). TIVA, total intravenous anesthesia; VNRS, verbal numerical rating scale; POD, postoperative day.

### Analgesics administration with intravenous PCA and rescue analgesics requirements

Although the morphine equivalent dose (mg) infused via IV PCA was smaller in TIVA group compared to Desflurane group during the postoperative 12–24 h interval, the overall dose used up to postoperative 48 h was comparable between the two groups (132.1 (108.7, 153.9) vs. 144.1 (126.0, 163.0), P = 0.235). Similarly, the number of bolus attempts were significantly smaller in the TIVA group compared to the Desflurane group at postoperative 0–12 h and 12–24 h periods but comparable for the overall postoperative 48 h (Table 4). There was no difference between groups in the amount of rescue analgesics administered up to 48 h after surgery.

**Table 4:** Data on intravenous patient-controlled analgesia.

	Desflurane group (n = 33)	TIVA group (n = 35)	*P* value
Morphine equivalent dose (mg)			
0–12 h	41.0 (36.9, 46.4)	36.9 (29.0, 43.8)	0.085
12–24 h	38.0 (33.2, 43.6)	32.8 (27.4, 40.1)	0.029
24–36 h	32.7 (28.5, 38.2)	31.4 (25.3, 38.5)	0.724
36–48 h	33.3 (26.9, 37.8)	32.2 (25.1, 36.9)	0.545
0–48 h	144.1 (126.0, 163.0)	132.1 (108.7, 153.9)	0.235
Bolus attempts			
0–12 h	18.5 (9.8, 27.3)	12.0 (3.0, 20.0)	0.024
12–24 h	11.0 (5.8, 16.0)	5.0 (0.0, 12.0)	0.010
24–36 h	5.0 (0.8, 8.3)	7.0 (0.0, 10.0)	0.690
36–48 h	4.0 (0.0, 8.5)	3.0 (0.0, 10.0)	0.529
0–48 h	43.5 (25.8, 56.5)	28.0 (8.0, 50.0)	0.056

Data are presented as median (interquartile range). TIVA, total intravenous anesthesia.

## Discussion

While inhalational anesthesia is the most used and conventional method of general anesthesia, the popularity of propofol-based intravenous anesthesia has grown in recent years. Behind this growing interest are several reasons, one of which is the possibility that TIVA is able to increase patients’ satisfaction after surgery under general anesthesia [20]. In the current study, we found that global QoR-40 scores were not influenced by type of general anesthesia after corrective lower limb osteotomy. However, among the five subdimensions of the QoR-40 questionnaire, TIVA was found to improve postoperative physical independence compared to desflurane anesthesia. Although scores of the subdimension of pain in the QoR-40 questionnaire was not different, patients of the TIVA group made fewer bolus attempts via IV PCA compared to the patients of the Desflurane group without any difference in additional rescue analgesics administration. TIVA was also able to significantly decrease nausea scores and rescue antiemetic requirements at the PACU.

QOR after surgery goes beyond the traditional analysis of morbidity and mortality, and the importance of patient-reported outcome measures are being increasingly recognized [21]. The QoR-40 scale is among the most extensively studied scoring systems that provide a quantitative measure of QOR after surgery and anesthesia [13]. We found global QoR-40 scores on POD 1 to be higher in the TIVA group compared to the Desflurane group, but the difference did not reach statistical significance. However, the difference in means between groups is 11.1, which exceeds the minimal clinically important difference (MCID) for the QoR-40. MCID is defined as the smallest change in score that reflects a meaningful change in health status, and perioperative interventions that result in change of 6.3 or more in QoR-40 scores signify a clinically important improvement or deterioration [22]. Moreover, the better outcome in the dimension of physical independence on POD 1 and 2 in the TIVA group is meaningful in that this score can be associated with early resumption of daily activities [19] which in turn may possibly predict better long-term quality of life [23]. The dimension of physical independence includes abilities to return to work, write, speak, wash, and look after one’s own appearance. These results are significant, considering the growing interest in early recovery after surgery [24] and the importance of early mobilization and functional recovery after orthopaedic surgery [25–27].

It is noteworthy that the preoperative global QoR-40 scores in our study population were somewhat lower than that reported in a previous study based on patients undergoing thyroidectomy for thyroid neoplasms [2]. This may seem counterintuitive considering the relatively younger and physically healthier patient population of our study. However, reasons for undergoing corrective lower limb osteotomy varies from occupational need, family opinion, peer pressure, ability to do certain sports or activities, and psychological mood [28,29]. The need to improve everyday function, reduce pain, and also improve psychological well-being through surgical correction are important components of the expectations that patients may have before undergoing corrective osteotomies. For patient populations that share similar aspects in terms of the patients’ expectations and general sense of well-being, the results of the present study may be able to shed some light on the perioperative management of such patients.

One well-known advantage of using propofol TIVA over inhalational anesthesia is the ability of propofol to reduce PONV [20,30,31]. Nausea, vomiting, and retching is included in the dimension of physical comfort of QoR-40, where ease of breathing, ability to have good sleep and enjoy food, feeling rested or restless, presence of shaking, twitching or shivering, and feeling too cold or dizzy are also included. However, the outcomes of this dimension in previous studies that compared propofol-based TIVA and inhalational anesthetics are inconsistent [2–8], and we were not able to find any improvement in scores with TIVA in our study. Although VNRS scores for nausea were found to be significantly lower in the TIVA group compared to the Desflurane group at the PACU, there was no difference in nausea scores, incidence of vomiting/retching, or the use of antiemetics on POD 1 and 2. These results are similar to previous studies where the antiemetic effect of propofol TIVA was found to be limited to the early postoperative period [32,33].

In terms of the dimension of pain, we were not able to see a significant difference between the two groups throughout the study. However, in a single question assessing the presence of severe pain, the score was higher in the TIVA group compared to the Desflurane group on both POD 1. In the same vein, a larger dose of analgesics via IV PCA was required during postoperative 12–24 h, and a significantly greater number of bolus attempts during postoperative 0–12 h and 12–24 h were observed in the Desflurane group compared to the TIVA group. The dimension of pain of the QoR-40 questionnaire not only includes surgical site pain, but also pain in extrasurgical sites such as headache, muscle pains, backache, sore throat, and sore mouth. The patient’s perception of “severe pain” and the use of analgesics to alleviate such pain may not align with the scores in the dimension of pain in QoR-40, and therefore, the results of the present study should be interpreted with caution. It should be noted however, that in previous studies, propofol-based TIVA was found to improve postoperative pain compared to inhalational anesthesia [20,31].

Compared to inhalational anesthetics, propofol has been shown to suppress the neuroendocrine stress response and reduce the release of catecholamines and cortisol during the perioperative period [34]. Propofol was also found to inhibit the release of pro-inflammatory cytokines, improve anti-inflammatory cytokine release and attenuate hyperglycemia during surgery [35,36]. The favorable results seen in the TIVA group of the present study may be attributable to these aforementioned characteristics of propofol.

There are several limitations to this study. First, sample size calculation was based on the difference of the global QoR-40 scores, and therefore was not sufficient to determine the difference in individual domains of the questionnaire. Second, we did not include long-term functional outcome or quality of life as outcomes in this study. While early postoperative QOR may be related to quality of life several months after surgery [23], we were not able to confirm this. Third, the patients enrolled in our study was relatively young and healthy and therefore it is difficult to generalize our results to a broader patient population undergoing surgery that may not share similar characteristics.

## Conclusions

In conclusion, we did not find any difference in global QoR-40 scores after corrective lower limb osteotomies between desflurane-based inhalational anesthesia and propofol-based TIVA. The more favorable scores in the dimension of physical independence in the TIVA group may suggest the possibility of better quality of life and resumption of daily activities in the early postoperative period [24–27]. The lesser presence of severe pain and the fewer PCA rescue bolus attempts may reflect a superior analgesic effect of TIVA compared to desflurane anesthesia. This together with its confirmed antiemetic effects in the early postoperative period, propofol TIVA should be considered as a useful component of anesthetic management in patients undergoing corrective lower limb osteotomies.

## Supporting information

S1 Checklist CONSORT checklist. (DOC)

S1 Data (XLSX)

S1 File Trial protocol (English). (DOCX)

S2 File Trial protocol (Korean). (DOCX)
