# Supplementary material for: Inhalational versus intravenous maintenance of anesthesia for quality of recovery in patients undergoing corrective lower limb osteotomy: A randomized controlled trial
Source: PLoS One. 2021 Feb 19;16(2):e0247089. doi: 10.1371/journal.pone.0247089 (PMC7894931; doi:10.1371/journal.pone.0247089)
Supplement: S1 File — (DOCX) [file pone.0247089.s003.docx]

**1. Project title**

Effect of anesthesia on quality of recovery in patients undergoing correctional tibial osteotomy – A randomized controlled trial

**2. Research objectives**

(1) To compare quality of recovery using the QoR-40 questionnaire between inhalational anesthesia and total intravenous anesthesia in patients aged 19 to 65 undergoing correctional tibial osteotomy.

(2) To compare vital signs at end of surgery, intraoperative BIS, intraoperative remifentanil requirements, time from anesthetic discontinuance to verbal response, duration of stay at post-anesthesia recovery unit, incidence of postoperative nausea and vomiting between inhalational anesthesia and total intravenous anesthesia in patients aged 19 to 65 undergoing correctional tibial osteotomy.

**3. Research site and trial duration**

- Site: Sinchon Severance Hospital
- Trial duration: 4 years from IRB approval

**4. Research subject inclusion/exclusion criteria and screening items**

- Inclusion criteria:

Patients scheduled for corrective tibial osteotomy including high tibial osteotomy and cosmetic lower limb lengthening to treat osteoarthritis, short stature or leg length discrepancy, between ages 19 and 65

- Exclusion criteria:
  - Patient refusal
  - Patients presenting with depressed mental status
  - Heart failure of ejection fraction less than 55%
  - Recent myocardial infarct or cerebral vascular accident within 1 year
  - Recent cardiac or major vessel surgery within 1 year
  - Reduced renal function (serum Cr>1.0mg/dL)
  - Patients with known hypersensitivity to propofol
  - Illiteracy
  - Patients with known Alzheimer’s disease or cognitive dysfunction
- Screening items: Past medical history, current medications, vital signs, complete blood count, coagulation test, blood chemistry test, electrocardiography, chest x-ray, urinalysis

**5. Sample size**

- The sample size of this study is 38 patients per group, total 76

- Sample size calculation was done under the hypothesis that a difference in global QoR-40 score of 10 or more between groups would be clinically significant. To obtain 90% power with a significance level of 5% by independent t-test and allowing for a dropout rate of 10%, 38 patients per group are needed.

**6. Methodology**

**1) Patient enrollment**

This study will be done as a prospective randomized trial. Patients aged 19 to 65 that are scheduled for corrective lower limb osteotomies including high tibial osteotomy and cosmetic lower limb lengthening, will be enrolled after obtaining informed consent from the patients and consulting with the attending orthopedic surgeon. The primary investigator will explain the study objective and methods to patients and obtain written informed consent at least 1 hour thereafter. Consent will be obtained at a private setting. Patients that have given consent to participate in this clinical trial will be screened and enrolled only when satisfying the inclusion/exclusion criteria. Each patient’s intent to participate in this trial will be checked and confirmed at the operating room before proceeding with study interventions.

**2) Interventions**

Patients will be randomly allocated to receive either desflurane-based general anesthesia (group D) or propofol-based total intravenous anesthesia (group P) on the day before surgery in a 1:1 ratio using a computer-generated random table generator.

All patients will maintain NPO for 8 hours before surgery. Upon arrival at the operating room, standard monitoring including pulse oximetry, non-invasive blood pressure monitoring, electrocardiography, and bispectral index (BIS, VISTA Monitoring System, Aspect Medical Systems Inc., Norwood, MA, USA) monitoring will be applied in all patients. In group D, general anesthesia will be induced with 5 mg kg^-1^ thiopental sodium and maintained with 4-7% desflurane and remifentanil infusion according to the Minto model to maintain a BIS value between 40 to 60. In group P, anesthesia will be induced and maintained with propofol and remifentanil using an effect-site target-controlled infusion pump (Orchestra Base Primea: Fresenius Vial, Brezins, France) according to the Marsh model (propofol) and Minto model (remifentanil). Rocuronium (0.6mg kg^-1^) will be used before intubation in all patients. Mechanical ventilation will be performed with 8 ml kg^-1^ of tidal volume and respiratory rates adjusted to maintain the end tidal CO_2_ levels between 35 to 45 mmHg with 50% oxygen/air mixture.

All patients will be given ramosetron 0.3 mg and fentanyl 1µg/kg at end of surgery. Concurrently, intravenous patient-controlled analgesia (IV PCA) consisting of fentanyl 0.2 µg kg^-1^ ml^-1^ and ramosetron 0.3 mg (total volume including normal saline, 150 ml) will be initiated at a 2 ml h^-1^ (0.4 µg kg^-1^ h^-1^) background infusion rate and 0.5 ml (0.1µg kg^-1^) on-demand bolus dose with a 15 min lockout time. Postoperative analgesia will consist of tramadol, acetaminophen, and meperidine given as needed at the orthopedic surgeon’s discretion.

**3) Observations**

QOR will be assessed by using the QoR-40 questionnaire at three time-points: the day before surgery, POD 1 and POD 2 in the evening on 7 pm. The QoR-40 is a 40-item questionnaire that is comprised of five dimensions; physical comfort (12 items), emotional state (9 items), physical independence (5 items), psychological support (7 items), and pain (7 items). Each item is graded on a five-point Likert scale, and global QoR-40 scores range from 40 (extremely poor QOR) to 200 (excellent QOR).

Intraoperative heart rate, mean blood pressure, and BIS will be recorded at baseline, 10 min after induction, cessation of anaesthetics, and at tracheal extubation. Response time is defined as time taken from cessation of anaesthetics to clear verbal response from the patient. Total intraoperative remifentanil and propofol dose will be recorded and compared between groups. Vital signs at the PACU will collected at admission and discharge.

Nausea scores will be evaluated at the PACU (highest score during the stay), POD 1 and POD 2 (same time point of QoR-40 assessment) using an 11-point verbal numerical rating scale (VNRS) ranging from 0=no nausea to 10=worst imaginable nausea. The incidence of vomiting and the amount of rescue antiemetics (metocloprimide 10mg or ramosetron 0.3mg) administered upon patient request will also be recorded. The cumulative amount of analgesics administered, and number of bolus attempts via IV PCA up to 48 h after surgery will be recorded and analyzed at 12h intervals. The number of patients requiring rescue analgesics up to postoperative 48h will be assessed and compared between groups.

**4) Data management and analysis**

Patient records will be managed by the primary investigator, professor Seokyung Shin. To protect patient information, enrolled subjects will be identified by initials and case numbers. Case report forms will be stored in a safe with a lock device. All data will be stored in a PC with limited access. All results will be presented as mean ± SD, median (IQR) or number (proportion) as appropriate. Fisher’s exact test will be used to analyze categorical variables and continuous variables will be analyzed with the independent t-test and paired t-test. P-values lower than 0.05 will be considered statistically significant. All statistical analyses will be performed using SPSS Statistics (IBM Corp., Armonk, NY, USA).
