# Supplementary material for: Inhalational versus intravenous maintenance of anesthesia for quality of recovery in patients undergoing corrective lower limb osteotomy: A randomized controlled trial
Source: PLoS One. 2021 Feb 19;16(2):e0247089. doi: 10.1371/journal.pone.0247089 (PMC7894931; doi:10.1371/journal.pone.0247089)
Supplement: S2 File — (DOCX) [file pone.0247089.s004.docx]

**1. 연구 제목**

한글: 경골 교정 절골술 (correctional tibial osteotomy)을 시행 받는 환자에서 흡입마취와 전정맥마취 간의 회복의 질 비교

영문: Effect of anesthesia on quality of recovery in patients undergoing correctional tibial osteotomy – A randomized controlled trial

**2. 연구 목적**

(1) 전신 마취 하에서 경골 교정 절골술을 받는 19세 이상 65세 이하 성인 환자에서 QoR-40 questionnaire를 통해 흡입마취와 전정맥마취 간의 회복의 질을 비교한다.

(2) 전신 마취 하에서 경골 교정 절골술을 받는 19세 이상 65세 이하 성인 환자에서 기관 발관시의 생체 징후, 수술 중 이중분광계수 (bispectral index), 수술 중 사용된 remifentanil의 용량, 마취 약제 사용 중단 시점에서 의사 소통 가능 시점까지의 시간, 회복실 체류 시간, 수술 후 오심구토 발생 등을 통해 흡입마취와 전정맥마취 간의 회복의 질을 비교한다.

**3. 연구 수행장소 및 기간**

- 실시기관: 신촌 세브란스 병원
- 연구기간: IRB 승인 시점부터 4년

**4. 대상자의 선정 또는 제외기준 및 스크리닝 검사 항목**

- 대상자 선정:

골관절염 (osteoarthritis), 단신 (short stature) 또는 다리길이 불일치 (leg length discrepancy)로 경골 교정 절골술 (correctional tibial osteotomy)에 속하는 근위경골절골술 (high tibial osteotomy)과 다리 길이 연장술 (cosmetic lower limb lengthening)을 시행 받는 19세 이상 65세 이하 환자

- 제외기준:
  - 환자가 연구 참여를 거부한 경우
  - 의식 수준이 저하되어 있는 환자
  - 좌심실 박출률 <55%의 심부전
  - 최근 1년 이내의 심근 경색, 뇌졸중
  - 최근 1년 이내의 심장 수술 등의 대혈관 수술 기왕력
  - 신장 기능 저하 환자 (serum Cr>1.0mg/dL)
  - Propofol에 대한 과민성이 있는 환자
  - 문맹 또는 외국인 환자
  - 치매 또는 인지 장애 환자
- 스크리닝 검사 항목: 과거력 및 병력, 투약 내역, 생체 징후, 일반 혈액 및 혈액 응고 검사, 일반화학 검사, 심전도, 흉부 엑스레이, 소변 검사

**6. 목표 대상자의 수 및 산출 근거**

- 목표 대상자 수는 군당 38명, 총 76명으로 한다.

- QoR-40 score의 차이가 10인 경우, 회복의 질은 15%의 차이가 있다. Sample size는 QoR-40 score의 차이가 10 이상인 경우 임상적으로 의미 있다는 가정하에 alpha 0.05, 90% power에서 각 군34명이 산출되며 중도 탈락율을 약 10% 로 고려하여 각 군당38명이 산출되었다.

**7. 연구 설계 및 방법**

**1) 연구 대상자의 등록**

본 연구는 전향적으로 진행하며, 하지의 기능적 및 외형적 교정을 위해 본원에서 시행 되는 교정적 경골 절골술에 속하는 두 가지 수술인 근위경골절골술 (high tibial osteotomy)과 다리 길이 연장술 (cosmetic lower limb lengthening)을 시행 받는 19세 이상 65세 이하 환자에 대해 정형외과 주치의의 동의를 얻은 후 연구 등록 여부를 결정한다. 연구 책임자가 환자에게 연구목적과 방법에 대해 설명한 후 최소 1시간 후 재방문 하여 동의 의사를 확인한 후 서면 승낙을 받는다. 연구 목적에 대한 설명과 서면 승낙은 병동의 독립된 상담실에서 실시한다. 연구 참여에 동의한 대상자를 스크리닝하여 선발 기준을 만족하는 경우 최종적으로 연구에 등록하며, 수술실에 입실하면 참여 의사를 재확인한 후 연구를 진행한다.

**2) 주술기 관리**

컴퓨터 생성 난수표를 이용하여 수술 전날에 연구 대상자를 무작위로 두 군으로 나누어 desflurane 전신마취(group D), propofol 전정맥 전신마취 (group P)로 마취를 시행하도록 한다. 환자들은 최소 8시간 금식한 상태에서 수술실에 입실한다. 수술실 입실 후 환자 감시를 위해 심전도, 비침습적 혈압계, 맥박 산소 포화도 감시기(pulse oximetry, SpO_2_), bispectral index (BIS, VISTA Monitoring System, Aspect Medical Systems Inc., Norwood, MA, USA) 모니터를 부착한다. Group D의 환자는 5 mg kg^-1^의 thiopental sodium으로 전신마취를 유도한다. 수술 중 마취는 oxygen-air 혼합가스에(50:50) 호기말 4~7% desflurane 과 Minto model에 따른 remifentanil infusion으로 BIS 점수를 40-60으로 유지한다. Group P의 환자는 Marsh model (propofol)과 Minto model (remifentanil)에 따라 목표농도조절주입장치 (Orchestra Base Primea: Fresenius Vial, Brezins, France)를 이용하여 마취를 유도 및 유지한다. 모든 환자에게 기관 삽관 전 rocuronium (0.6mg kg^-1^)을 투여한다. 기계 환기는 8ml/kg의 tidal volume으로 호흡 수를 조절하여 end tidal CO_2_를 35~45mmHg로 조절한다.

모든 대상자는 수술 종료 시점에 ramosetron 0.3mg과 fentanyl 1mcg/kg를 투여받는다. 정맥 자가 통증 조절 (PCA)은 0.2mcg/kg/ml의 fentanyl, ramosetron 0.3mg과 생리식염수를 사용하여 총 용량 150ml으로 하며, basal rate 0.2 ml/h, bolus dose 0.5 ml, lockout time 15분으로 설정한다. 모든 환자는 수술 후 추가적인 통증 조절이 필요할 경우 정형외과 주치의의 판단에 따라 tramadol, acetaminophen, meperidine을 정맥 투여한다.

**3) 관찰**

모든 대상자에서 QoR-40 questionnaire는 수술 전, 수술 후 1일과 2일 오후 7시에 (총 3회) 시행한다. QoR-40 questionnaire는 회복의 다섯 가지 측면을 측정한다: physical comfort (12 items), emotional state (9 items), physical independence (5 items), psychological support (7 items), pain (7 items). 각 item은 5점의 Likert scale (전혀, 가끔, 보통, 대부분, 항상)으로 분류되며, 총점은 40점 (최악의 회복의 질)부터 200점 (최상의 회복의 질)까지 분포한다.

주술기의 맥박과 평균 혈압, BIS는 마취 유도 전, 마취 유도 10분 후, 주 마취약제 사용 중단시와 기관 발관시에 기록한다. 반응 시간은 주마취약제 중단 시점으로부터 명확한 verbal response를 보인 시점까지의 기간으로 정의한다. 수술 중 사용된 remifentanil 및 propofol의 용량을 기록하여 군간 비교한다. 회복실에서는 회복실 도착 및 퇴실시의 혈압과 맥박, 회복실 체류 시간을 기록한다.

오심 점수는 회복실 (체류 중 최고 점수)과 수술 1, 2일 후 (QoR 평가와 같은 시점)에 11-point verbal numerical rating scale을 통해 기록한다 (0= 오심 없음, 10= 최악의 오심), 구토 발생 및 환자의 요구에 따라 투여된 항구토제 사용량 (metocloprimide 10mg 또는 ramosetron 0.3mg)을 기록한다. 수술 후 48시간까지의 정맥 PCA의 사용량과 bolus attempt 횟수를 기록하고 12 시간 단위로 분석한다. 수술 후 48시간까지 사용한 rescue analgesics 투여량를 평가하여 군간 비교한다.

**4) 데이터 관리 및 분석**

환자의 자료는 본 실험의 연구 책임자인 신서경 교수가 관리한다. 대상자의 정보가 드러나지 않도록 식별 정보는 영문 이니셜과 케이스 번호로 코드화 하고 증례 기록서는 잠금 장치가 있는 금고에 보관하며 정리된 데이터는 접근이 제한된 컴퓨터에 저장한다. 모든 결과는 평균±표준편차, 중간값(사분위간변위 (1사분위수-3사분위수)) 또는 환자의 숫자 (비율)로 표시한다. 범주형 자료는 Fisher’s exact test로 군간 비교하며, 연속형 자료는 independent t-test 및 paired t-test로 군간 비교할 계획이다. P-value가 0.05 이하인 경우, 통계적으로 유의한 것으로 간주하며, 모든 통계분석은 SPSS Statistics (IBM Corp., Armonk, NY, USA)를 사용한다.
